# Development and evaluation of a social inclusion framework for a comprehensive hospital-based elder abuse intervention

**DOI:** 10.1371/journal.pone.0234195

**Published:** 2020-06-05

**Authors:** Janice Du Mont, S. Daisy Kosa, Hannah Kia, Charmaine Spencer, Mark Yaffe, Sheila Macdonald

**Affiliations:** 1 Women’s College Research Institute, Women’s College Hospital, Toronto, Ontario, Canada; 2 Dalla Lana School of Public Health, University of Toronto, Toronto, Ontario, Canada; 3 Ontario Network of Sexual Assault/Domestic Violence Treatments Centres, Toronto, Ontario, Canada; 4 School of Social Work, University of British Columbia, Vancouver, British Columbia, Canada; 5 Gerontology Research Centre, Simon Fraser University, Vancouver, British Columbia, Canada; 6 Department of Family Medicine, McGill University, Montreal, Québec, Canada; 7 Department of Family Medicine, St. Mary’s Hospital Centre, Montreal, Québec, Canada; Universidade Estadual Paulista Julio de Mesquita Filho Faculdade de Medicina Campus de Botucatu, BRAZIL

## Abstract

A framework of social inclusion can promote equity and aid in preventing and addressing the abuse of older adults. Our objective was to build a social inclusion framework for a comprehensive hospital-based elder abuse intervention being developed. Potential components of such a framework, namely, health determinants and guiding principles, were extracted from a systematic scoping review of existing responses (e.g., interventions, protocols) to elder abuse and collated. These were subsequently rated for their importance to the elder abuse intervention by a panel of violence experts and further evaluated by a panel of elder abuse experts. The final social inclusion framework comprised 12 health determinants each representing factors underpinning susceptibility for abuse in aging populations: history of trauma/abuse, communication needs, disability, health status, mental capacity, social support, culture, language, sexuality, religion, gender identity, and socioeconomic status. The framework also comprised 19 guiding principles each encompassing considerations for equitable engagement with older adults (e.g., All older adults have the right to self-determination, All older adults have the right to be safe, All older adults are assumed competent unless determined otherwise). Integrating this social inclusion framework into the design and delivery of an elder abuse intervention could empower older adults, while at the same time ensuring that practices and policies are tailored to meet their unique and varying needs.

## Introduction

Elder abuse, alternatively referred as the abuse or neglect of older adults, is increasingly acknowledged as a pervasive societal and health problem [1, 2]. According to Justice Canada, abuse of older adults “refers to violence, mistreatment or neglect that older adults living in either private residences or institutions may experience at the hands of their spouses, children, other family members, caregivers, service providers or other individuals in situations of power or trust. The definition also includes older adults abused by non-family members who are not in a position of power or trust” [3], p.1. In a 2017 systematic review of prevalence of elder abuse in community settings globally, the pooled prevalence rate for overall elder abuse, which included physical, sexual, emotional, financial abuse, and/or neglect, was 15.7% [4]. The Centers for Disease Control and Prevention [5] has reported that older adults who experience abuse may sustain immediate physical injuries, suffer serious morbidity, including psychological distress, depression, and sleep disturbances, and be at increased risk of premature death. Elder abuse has also been associated with increased use of health services, including emergency department visits and stays in hospital [6].

Elder abuse may be caused by, or result in, experiences of social isolation [7, 8]. Social isolation is commonly defined as an individual’s or group’s lack or low quantity and quality of social contact with others in the community and institutions [7, 9, 10]. It is estimated that up to 20% of older adults in Canada currently experience some degree of social isolation [8]. Older adults experiencing social isolation are at an increased risk of adverse physical (e.g., serious falls, stroke, coronary heart disease) and psychological (e.g., depression, sleep disturbance) outcomes [11, 12], which in turn, may heighten their vulnerability to experiences of elder abuse [6]. Therefore, there is a need to recognize and account for social isolation in preventing and addressing abuse among older adults [8].

### Conceptualization of social inclusion as a framework for addressing elder abuse

Social isolation at the interpersonal level is often conceptualized as being closely associated with–and sometimes indicative of–the more systemic phenomenon of social exclusion in older adults [9, 13]. Social exclusion is characterized by social isolation that occurs as a result of factors beyond the individual’s or group’s control [9] and has been linked to inequitable access to “resources, capabilities and rights which leads to health inequalities” [14], p.2. Older adults are most prominently excluded in the contexts of social relations, material and financial resources, civic participation, and services and amenities, among others [13] and specifically, with regard to decisions in the workplace, within families and communities, and in medical settings [15, 16]. Theories of social exclusion have been used to foreground and identify issues of inequity reflected in the social conditions and experiences of older adults and, by extension, inform prevention and intervention strategies to enhance the health and well-being of aging populations [17, 18]. Indeed, these theories have highlighted the relevance of constructing and using social inclusion frameworks across diverse areas of policy and practice to mitigate inequities affecting older adults and, in turn, systematically aid in preventing and addressing adverse health outcomes associated with social exclusion in aging populations [17, 18].

The use of a social inclusion framework in the elder abuse context offers the advantage of considering not only the impact of age on abuse, but other key determinants of health such as gender, ethnicity, disability, socioeconomic status, social support, and trauma history and how these intersect with relational and structural disadvantage [19, 20]. Determinants of health have been highlighted in the literature for their role in influencing older adults’ access to a variety of resources required for equitable social engagement and, therefore, predicting social inclusion [13]. As such, a comprehensive social inclusion framework for elder abuse needs to account for health determinants that may be particularly salient in fostering conditions of social exclusion and in placing an older adult at a heightened risk of experiencing one or more types of abuse [20–22]. These and other related factors may additionally create barriers to disclosing abuse or accessing care, as well as affect the receipt of that care by the older adult [23–25].

In addition to accounting for multiple determinants of health, a social inclusion framework for addressing elder abuse needs to incorporate principles that ensure an intervention is empowering and used equitably. Indeed, as ageism and age-based discrimination contribute to various forms of social exclusion in older age [26], it has been increasingly acknowledged that it is important to integrate key principles or values of engagement in any framework of social inclusion used to prevent and address the abuse of older adults [19, 20, 27].

Recognition of relevant health determinants, along with the integration of key guiding principles for engagement with older adults, can inform the development of a framework of social inclusion used for preventing and addressing the abuse of older adults [19, 20]. The utilization of a social inclusion framework in the development of policies and practices can combat discrimination against older adults, promote access to and receipt of appropriate services to improve their health and well-being and, ultimately, enable their full participation in the community [28, 29].

### The present study

The goal of our study was to gain insight on the most salient health determinants and guiding principles to incorporate into the development of a social inclusion framework using available evidence and expert opinion. This study is part of a larger multi-phase, multi-method program of research being conducted in collaboration with the Ontario Network of Sexual Assault/Domestic Violence Treatment Centres (SA/DVTCs) to develop, implement, and evaluate an elder abuse intervention [30, 31]. This intervention will be built on the infrastructure of Ontario’s sexual assault nurse examiner-led hospital-based SA/DVTCs with formalized links to the allied health, legal, and community sectors [30, 31]. These centres provide 24/7 acute care to women, men, children, and trans persons of all ages who have experienced a sexual assault and physical assault by an intimate partner, including crisis intervention, medical assessment and treatment, documentation of injuries, collection of forensic evidence, risk assessment, safety planning, follow-up medical care and counselling, and referral to various community agencies for other forms of support (e.g., legal counsel, housing) [32]. However, across these centres, there is no standardized provision of dedicated care for all types of elder abuse. The elder abuse intervention under development is designed to address sexual, physical, and psychosocial abuse and neglect of older adults who may walk in or be referred to these treatment centres by emergency departments in hospitals, other health professionals (e.g., family physicians), or community and social services (e.g., police, long-term care institutions). The social inclusion framework developed in this study will help guide the implementation and evaluation of the finalized elder abuse intervention.

## Methods

Three steps were involved in the development of the social inclusion framework for the elder abuse intervention under development: 1) A list of potential components, namely any health determinant and guiding principle, were first identified in a systematic scoping review of the grey and scholarly literatures [30]; 2) This list of potential components was then evaluated for their relevance and importance to the social inclusion framework of the elder abuse intervention under development by a panel of SA/DVTC program leaders [33]; and 3) Finally, the list of draft components were evaluated by a panel of key stakeholders with expertise in the abuse of older adults. This study was reviewed by Women’s College Hospital’s Research Ethics Board (REB) in Toronto, Ontario; the SA/DVTC panel, which was part of a larger consensus process to develop the elder abuse intervention (REB Number 2013-0059-E) [31, 33] and the key stakeholders in elder abuse panel (REB Number 2015-0096-E).

### Systematic scoping review to extract potential components of a social inclusion framework

In this step, we extracted possible components of a social inclusion framework from 68 responses (e.g., interventions, protocols, guidelines) containing recommendations relevant to a multidisciplinary and intersectoral hospital-based elder abuse intervention. The comprehensive search strategy, pre-determined eligibility criteria, and selection process employed, as well as other data extracted, are described in detail in Du Mont et al. (2015) [30]. The extraction and collation of the components of the social inclusion framework described in this study has not been previously published.

#### Data extraction and collation

Four reviewers (JDM, SE, SM, MW) with expertise in conducting systematic scoping reviews, elder abuse, gender-based violence, and health systems innovation and evaluation, piloted a data extraction form that included health determinants and guiding principles, each section with an option to list other items not captured. These reviewers extracted data independently from five responses and then met to review any inconsistencies and clarify items on the form where necessary [30]. After revision of the data collection form, two reviewers (SE, SDK) recorded the potential components of the social inclusion framework, namely any health determinant and guiding principle, explicitly addressed from the 68 responses. Disagreements in extraction were resolved, where consensus could not be reached between the two reviewers, by a senior research team member (JDM). If responses addressed the component conceptually or were variations of it, the item was recorded as included (e.g., considerations of woman and man were recorded as gender).

Items in the ‘Other’ categories were collated into components (SE, SDK, SM, JDM). For example, ‘disability’, ‘disabled’, ‘functional disabilities’, ‘developmentally disabled’ were collated as ‘disability’. Although a type of disability, specific reference to communication needs (e.g., ‘verbally unable to communicate’, ‘communication impairment’) was collated separately from disability, because of the salience of communication needs in working with older adults [34, 35]. Guiding principles collated included: The rights of older adults supersede the organization's/provider's personal interests, Do no harm, Older adults are never to be held responsible for the abuse that they have experienced, Any intervention should empower the older adult, All older adults have the right to clear education/information on elder abuse.

The resulting list of potential components of a social inclusion framework included 11 health determinants: ‘health status’ (includes mental and physical health), ‘mental capacity’ (e.g., to consent to care), ‘social support’, ‘culture’ (includes Indigenous status), ‘socioeconomic status’ (includes educational background), ‘gender’, ‘religion’, ‘language’ (includes inability to speak the language of the service provider or speaks the local language as a second language), ‘disability’ (includes cognitive and physical disabilities), ‘sexual orientation’, and ‘communication needs’; see Table 1); and 19 guiding principles (e.g., ‘All older adults have the right to be safe’, ‘All older adults are assumed competent unless determined otherwise’; see Table 2).

**Table 1:** Identification and evaluation of health determinants for a social inclusion framework for an elder abuse intervention.

Systematic Scoping Review	Sexual Assault/Domestic Violence Treatment Centre Program Leader Panel (n = 12)			Key Stakeholders in Elder Abuse Panel (n = 22)
Potential health determinant	Draft health determinant	Rating*		Final health determinant
		Mean	SD	
	History of trauma/abuse**	5.00	0.00	History of trauma/abuse
Communication needs	Communication needs	4.92	0.29	Communication needs
Disability	Disability	4.92	0.29	Disability
Health status	Health status	4.92	0.29	Health status
Mental capacity	Mental capacity	4.92	0.29	Mental capacity
Social support	Social support	4.92	0.29	Social support
Culture	Culture	4.83	0.39	Culture
Language	Language	4.75	0.45	Language
Sexual orientation	*Sexuality*	4.67	0.65	Sexuality
Religion	Religion	4.58	0.67	Religion
Gender	*Gender identity*	4.50	0.67	Gender identity
Socioeconomic status	Socioeconomic status	4.42	1.24	Socioeconomic status

SD = Standard deviation. Health determinants are listed in descending order of their mean Likert rating. Italics denote a change in the determinants.

*Likert scale: 1 = strongly disagree, 2 = somewhat disagree, 3 = neither disagree nor agree, 4 = somewhat agree, 5 = strongly agree

**Rated by three program leaders.

**Table 2:** Identification and evaluation of guiding principles for a social inclusion framework for an elder abuse intervention.

Systematic Scoping Review	Sexual Assault/Domestic Violence Treatment Centre Program Leader Panel (n = 12)			Key Stakeholders in Elder Abuse Panel (n = 22)
Potential guiding principle	Draft guiding principle	Rating*		Final guiding principle
		Mean	SD	
All older adults have the right to self-determination (e.g., accept/refuse services, make their own decisions)	All older adults have the right to self-determination (e.g., accept/refuse services, make their own decisions)	5.00	0.00	All older adults have the right to self-determination (e.g., accept/refuse services, make their own decisions)
All older adults have the right to be safe	All older adults have the right to be safe	5.00	0.00	All older adults have the right to be safe
All older adults are assumed competent unless determined otherwise	All older adults are assumed competent unless determined otherwise	5.00	0.00	All older adults are assumed competent unless determined otherwise
All older adults have the right to appropriate protection (when older adult is not competent)	All older adults have the right to appropriate protection	4.92	0.29	All older adults have the right to appropriate protection
All older adults have the right to dignity	All older adults have the right to dignity	4.92	0.29	All older adults have the right to dignity
All older adults have the right to retain civil and constitutional rights (unless restricted by courts)	All older adults have the right to retain civil and constitutional rights (unless restricted by courts)	4.92	0.29	All older adults have the right to retain civil and constitutional rights (unless restricted by courts)
Elder abuse is a complex issue	Elder abuse is a complex issue	4.92	0.29	Elder abuse is a complex issue
Any intervention should empower the older adult	Any intervention should empower the older adult	4.92	0.29	Any intervention should empower the older adult
All older adults have the right to clear education/ information on elder abuse	All older adults have the right to clear education/ information on elder abuse	4.92	0.29	All older adults have the right to clear education/ information on elder abuse
All older adults have the right to informal and formal support	All older adults have the right to informal and formal support	4.83	0.39	All older adults have the right to informal and formal support
All older adults can make decisions that do not conform to social norms if no harm is done to others	All older adults can make decisions that do not conform to social norms if no harm is done to others	4.79	0.40	All older adults can make decisions that do not conform to social norms if no harm is done to others
There is an ethical responsibility to identify elder abuse and address it whether deliberate or inadvertent	There is an ethical responsibility to identify elder abuse and address it	4.75	0.45	There is an ethical responsibility to identify elder abuse and address it
All older adults have the right to privacy and confidentiality	All older adults have the right to privacy and confidentiality	4.75	0.45	All older adults have the right to privacy and confidentiality
Older adults are never to be held responsible for the abuse that they have experienced	Older adults are never to be held responsible for the abuse that they have experienced	4.67	0.49	Older adults are never to be held responsible for the abuse that they have experienced
All care providers to older adults must be respectful of existing relationships	All care providers to older adults must be respectful of existing relationships	4.58	0.67	All care providers to older adults must be respectful of existing relationship
All care should focus on older adults’ best interest/improving their quality of life	All care should focus on older adults’ best interest/improving their quality of life	4.58	1.16	All care should focus on older adults’ best interest/improving their quality of life
Do no harm	Do no harm	4.50	1.17	Do no harm
The rights of older adults supersede the organization's/provider's personal interests	The rights of older adults supersede the organization's/provider's personal interests	4.17	1.19	The rights of older adults *should* supersede interests *of an organization*
				The rights of older adults *should* supersede *providers’ personal interests*
Many types of elder abuse are criminal offences	Many types of elder abuse are criminal offences	3.08	1.31	**

SD = Standard deviation. Guiding principles are listed in descending order of their mean Likert rating. Italics denote a change in the principle.

*Likert scale: 1 = strongly disagree, 2 = somewhat disagree, 3 = neither disagree nor agree, 4 = somewhat agree, 5 = strongly agree.

**Item removed based on mean Likert rating.

### Evaluation of extracted components of the social inclusion framework by panel of SA/DVTC program leaders

In this step, the potential components of the social inclusion framework were evaluated for their importance for framing the elder abuse intervention under development in a one-day meeting held in Toronto, Ontario on May 27, 2015 with a panel of SA/DVTC program leaders [33]. These panel members were selected based on their extensive clinical experience in responding to sexual assault and domestic violence survivors of all ages and genders, geographical representation of the province, and the diversity of the populations served by their centres (e.g., Indigenous peoples, Franco-Ontarians). They also were chosen as they are the key knowledge users who would ultimately oversee the implementation of the elder abuse intervention for which the social inclusion framework is being developed [33]. The number of panel members was limited to 12 in this meeting to allow for active discussion.

In the meeting, an update of the intervention was given and the importance of a social inclusion framework was explained. The extracted potential components of the social inclusion framework were presented. The panel was then asked to independently rate each health determinant and guiding principle on a paper-based questionnaire for its importance to the social inclusion framework under development, using a Likert scale (1 = strongly disagree, 2 = somewhat disagree, 3 = neither disagree nor agree, 4 = somewhat agree, 5 = strongly agree). Panel members were asked to note any changes they thought important to wording, as well as add any health determinants or guiding principles they believed to be missing and rate them on the same scale. A discussion of their responses was documented. Consent to participate was obtained as part of the larger consensus process to develop the elder abuse intervention [33].

#### Analysis

For each health determinant and guiding principle rated (including those written in), the mean and standard deviation of the Likert rating was calculated [36, 37]. The mean rating measured the average level of importance of each proposed item to the social inclusion framework (mean 4+ indicated important to very important). Those health determinants and guiding principles that achieved a mean rating of 4+ were retained for further consideration. Those that did not achieve this rating were dropped from the framework.

### Evaluation of draft components of the social inclusion framework by panel of key stakeholders in elder abuse

In this step, we reviewed, revised, and finalized the draft components of the social inclusion framework for the elder abuse intervention under development. A one day meeting was held on October 1, 2015, which brought together 22 intersectoral stakeholders with expertise and interest in elder abuse from Ontario, Alberta, and Quebec. This expert panel had additional expertise in geriatric medicine, gerontology, nursing and community care, sexual and domestic violence, family violence, crime prevention, and the law and represented the provincial government (e.g., Ministry of Citizenship and Immigration, Ontario Seniors Secretariat); national, provincial, and local networks (e.g., Canadian Network for the Prevention of Elder Abuse; Elder Abuse Ontario; North York Elder Abuse Network), health services (e.g., Renfrew Victoria Hospital, Brant Community Healthcare System), community organizations (e.g., Champlain Elder Abuse Response Coalition; Community Support Connections, Meals on Wheels and More; Family Service Toronto; Victim Services of Brant); and law enforcement (i.e., Halton Police Service). Also represented on the panel was a member of the public in their role as an older adult and consumer of health and social services. The selection of panel members was based on Government of Canada recommendations that frameworks to address the social isolation of older adults “be developed in consultation with seniors as well as key players from the not-for-profit, public and private sectors” [7], p.25. Additionally, panel members represented the various sectors that comprise the elder abuse intervention under development [31].

In the meeting, an overview of the Ontario Network of SA/DVTCs and their services was provided, followed by details on the development of the elder abuse intervention, including the steps taken to date on building its social inclusion framework. The draft health determinants and guiding principles were then presented and the panel was asked to review each, suggesting any changes in wording, as well as any components that might have been missed or should not be retained. The discussion was recorded on flipcharts by four research team members (SDK, SE, SL, SS) and the notes used to later refine the health determinants and guiding principles to comprise the social inclusion framework. The refined framework was then sent by email to several members of this panel who had agreed to do a final review of the components before the framework’s finalization. The need to obtain consent to participate in this review was waived by the REB.

## Results

### Draft components of the social inclusion framework

In the rating of the 11 health determinants by the SA/DVTC program leader panel, all received mean Likert ratings of 4+ (agreed or strongly agreed an item was important) (see Table 1). A new determinant was written-in on the questionnaire and rated 5 by three program leaders. During the discussion of responses, it was collated as ‘history of trauma/abuse’ and agreed to be an integral part of any social inclusion framework going forward. Additionally, the wording of ‘gender’ was revised to ‘gender identity’ and ‘sexual orientation’ to ‘sexuality’ (see supporting information file, S1 Table: Program Leader Panel Ratings of Components of a Social Inclusion Framework for an Elder Abuse Intervention).

Some minor suggestions for rewording of the 19 guiding principles were made by the program leader panel, however, all but one guiding principle rated received a mean Likert rating of 4+ (see Table 2). The principle that ‘Many types of elder abuse are criminal offences’ received a mean rating of 3.08 and, therefore, was dropped from consideration as a principle within the social inclusion framework.

### Final components of the social inclusion framework

The draft components of the social inclusion framework were reviewed by members of the panel of key stakeholders in elder abuse and except for the rewording of one principle were retained (see Tables 1 and 2). The guiding principle, ‘The rights of older adults supersede the organization's/provider's personal interests’, was split into two principles. With regard to these two principles, one panel member expressed the need to acknowledge that organizations and providers may be hampered by externally controlled operating regulations and funding and the safety, integrity, and best holistic outcomes for an older adult might necessitate some compromise.

## Discussion

In this study, we found that there was agreement that certain determinants of health, as well as several guiding principles, were salient components of a social inclusion framework for a hospital-based elder abuse intervention under development for future implementation and evaluation across Ontario. Twelve health determinants—rated highly by the panel of SA/DVTC program leaders and endorsed by the panel of key stakeholders in elder abuse—will form core components of the social inclusion framework. These health determinants include, in order of their deemed importance, ‘history of trauma/abuse’, ‘communication needs’, ‘disability’, ‘health status’, ‘mental capacity’, ‘social support’, culture’, language’, ‘sexuality’, ‘religion’, ‘gender identity’, and ‘socioeconomic status’. These determinants are consistent with many of those identified in the literature as relevant within frameworks to address the social isolation of older adults [7, 10], risk factors associated with one or more types of elder abuse [20–22, 38], and barriers to disclosing abuse and/or accessing information and services among older adults [23–25].

Importantly, the panel of SA/DVTC program leaders was instrumental in broadening and adding nuance to the list of determinants of health drawn from the elder abuse responses. The panel suggested rewording of the determinant ‘gender’, which has historically been comprised of the binary categories of ‘woman’ and ‘man’, to ‘gender identity’, a more inclusive term that better accounts for the impact of sociocultural influences on the behaviours and experiences of diverse persons. For example, there are many reported incidences of overt transphobia directed toward transgender older adult populations, which may increase their “resistan[ce] … to accepting [elder abuse] services, due to their fears of being victimized or ridiculed … and of losing especially-valued independence and privacy” [39], p. 46, [40, 41]. The refinement of ‘gender’ to ‘gender identity’ is in keeping with the continued emphasis globally on health equity and addressing gender diversity in all research, programs, and policy [42], as well as gendered disparities reflected in rates and experiences of various types of elder abuse [23, 38, 43–45].

The panel of program leaders also suggested the rewording of ‘sexual orientation’ to ‘sexuality’. ‘Sexual orientation’ is of known importance to consider when responding to elder abuse given the historical and contemporary marginalization, for example, of older lesbian, gay, and bisexual adults [39, 40, 46]. However, ‘sexuality’ acknowledges more broadly that older adults in general are often presumed to be asexual by service providers and sexual health [47]. Additionally, those with “expressions of non-normative sexuality and gender identity in old age” may be subjected to increased surveillance and control that limits their sexual freedom [48], p.1. Inclusion of ‘sexuality’, as a key component of the social inclusion framework, can promote the appropriate consideration of diverse sexualities and older adults’ sexual needs in the context of providing elder abuse care.

The program leader panel also identified ‘history of trauma/abuse’ as an important addition to the components of the social inclusion framework, which is consistent with studies that emphasize the salience of considering this factor in delivering health care for vulnerable populations [49]. Victimization in childhood, including childhood sexual abuse, has been associated with a greater likelihood of victimization in later life, including experiences of elder abuse [50]. Some older adults may experience abuse throughout their life course, especially those that co-habit with the abuser [51, 52]. Older adults with histories of abuse as well as those who have experienced trauma more generally (e.g., loss of family, friends, and children, life-threatening illnesses, injury, war and other conflicts) are at an increased likelihood of experiencing PTSD symptomology and other adverse health outcomes [51, 53, 54].

In addition to determining key determinants of health, there was general agreement between the two panels on the importance of various guiding principles as core components of a social inclusion framework. Two of the three most highly rated principles by program leaders—endorsed by the key stakeholders in elder abuse—‘All older adults have the right to self-determination’ and ‘All older adults have the right to be safe’ are consistent with principles listed in the 1991 United Nations Declaration on Aging and in other literature [27, 55]. The other most highly rated principle, ‘All older adults are assumed competent unless determined otherwise’, is protected in legislation across the globe, particularly in the United Kingdom, the United States, and Canada, and is well established in the medical community [55, 56]. Although not rated as highly, the principle ‘Older adults are never to be held responsible for the abuse that they have experienced’ may be of particular relevance to the training of health and social service providers, as one study of organizations that support survivors of elder abuse revealed that victim blaming may be common [57]. This principle has been applied successfully in the context of addressing sexual and domestic violence [58]. While it is important to understand the circumstances within which abuse has occurred, it is critical to the healing of all victims of violence to be treated in a non-judgmental and supportive manner [59].

The program leader panel revised two of the guiding principles removing conditions that had been placed on them. For the right to appropriate protection, the panel extended this right to all older adults all the time, not only when there is a concern about competence. Similarly, the panel did not feel that the ethical responsibility to respond to abuse needed to be in anyway affected by the intentions of the perpetrator (deliberate or inadvertent abuse). Another guiding principle, ‘Many types of elder abuse are criminal offences’, was rated less than important/very important and dropped. Only one principle, ‘The rights of older adults supersede the organization's/provider's personal interests,’ was revised (split into two) by the panel of key stakeholders in elder abuse, indicating that in their opinion the extracted, collated, and reviewed principles were considered integral to working with abused older adults.

The social inclusion framework developed in this study has been designed to promote equity by addressing systemic sources of social exclusion and isolation affecting aging populations. Indeed, given that the literature on inequity in aging has highlighted the relevance of drawing on principles of social inclusion to address diverse expressions of vulnerability rooted in the social exclusion and isolation of older adults [18], the current framework holds promise in accounting for elder abuse as a product of disadvantage. The framework will prompt relevant stakeholders (e.g., service providers) to consider determinants of health such as social support, culture, and socioeconomic status as indicators of social exclusion and, by extension, factors underlying the particularly heightened susceptibility of some older adults to experiences of elder abuse. Additionally, the framework’s guiding principles may aid these providers in translating the recognition of these factors into other policies that reflect the pursuit of social inclusion and greater equity for those affected by elder abuse.

The effectiveness of the elder abuse intervention under development in reducing social isolation, social exclusion, and elder abuse will need to be tested in future pilot research, including the uptake of the social inclusion framework within the policies and practices associated with the intervention. It should be acknowledged that in implementing the social inclusion framework as part of the pilot of the elder abuse intervention, there may be circumstances in which guiding principles may conflict with each other, as noted in discussion by the panel of key stakeholders in elder abuse, or with other components or legislative constraints associated with a hospital-based intervention. For example, the prioritization of an older adult’s safety as reflected in mandatory reporting requirements in some settings may conflict with their to right to self-determination, particularly if they do not consent to supportive involvement from relevant authorities and providers mandated to respond to elder abuse and neglect [60, 61]. Additionally, given the potential for organizational, regulatory, and financial restrictions to limit the range of possibilities for optimizing the care of older adults in situations of abuse, application of this framework may require flexibility and compromise by providers using it to enhance social inclusion for clients. While the social inclusion framework was developed for the Ontario context and, specifically, for a hospital-based intervention, it could inform other responses being developed elsewhere to address the abuse of diverse older adults.

### Limitations

There are several limitations in this study that are important to acknowledge. It is possible that since the initial scoping review was conducted, other recommendations have been made for social inclusion in the context of elder abuse responses. However, given the enduring nature of the principles and determinants of health extracted, it is very likely that they still hold relevance for the elder abuse intervention under development. Additionally, during the systematic scoping review, some potentially relevant components of a social inclusion framework may have been missed or not have been represented in the responses. However, both panels had opportunities to add any heath determinants and guiding principles they thought missing. Although the panels comprised highly experienced violence and elder abuse experts, many of whom are key knowledge users that will ultimately utilize the social inclusion framework in the implementation of the elder abuse intervention, expert opinion does not in and of itself constitute high level evidence. Moreover, differently composed panels may have produced different results.

## Conclusion

In this study, expert panels endorsed the utilization of a social inclusion framework in the development of an elder abuse intervention that promotes adherence to guiding principles and the examination of different life experiences and characteristics of all older adults to ensure that the care provided is delivered without the influence of individual and systemic biases [20]. The final social inclusion framework, comprised of 12 important health determinants and 19 principles, will underpin the training of a large and diverse range of service providers who will work within the elder abuse intervention [31], ensuring their competence to provide comprehensive care that is equitable and empowering. Ideally, the integration of this social inclusion framework in the design and delivery of the elder abuse intervention will serve older adults well by delivering care tailored to meet their unique and varying needs, while prioritizing and restoring respect for their rights, dignity, and freedoms [29, 62].

## Supporting information

S1 Table Program leader panel ratings of components of a social inclusion framework for an elder abuse intervention. (PDF)
