# Supplementary material for: Development and evaluation of a social inclusion framework for a comprehensive hospital-based elder abuse intervention
Source: PLoS One. 2020 Jun 5;15(6):e0234195. doi: 10.1371/journal.pone.0234195 (PMC7274390; doi:10.1371/journal.pone.0234195)
Supplement: S1 Table — (PDF) [file pone.0234195.s001.pdf]

S1 Table: Program Leader Panel Ratings of Components of a Social Inclusion Framework for an Elder Abuse Intervention

**Guiding Principle**	Expert 1	comments 1	Expert 2	comments 2	Expert 3	comments 3	Expert 4	comments 4	Expert 5	comments 5	Expert 6	comments 6
All older adults have the right to self-determination (e.g., accept/refuse services, make their own decisions)	5.0		5.0		5.0		5.0		5.0		5.0	
All older adults have the right to be safe	5.0		5.0		5.0		5.0		5.0		5.0	
All older adults have the right to privacy and confidentiality	5.0		5.0		5.0		5.0		5.0		4.0	
All older adults are assumed competent unless determined otherwise	5.0		5.0		5.0		5.0		5.0		5.0	
All older adults have the right to appropriate protection (when older adult is not competent)	5.0		5.0		5.0		5.0		5.0		5.0	
All older adults have the right to dignity	5.0		5.0		5.0		5.0		5.0		5.0	
All older adults have the right to retain civil and constitutional rights (unless restricted by courts)	5.0		5.0		5.0		5.0		5.0		5.0	
All older adults have the right to informal and formal support	5.0		5.0		5.0		5.0		5.0		4.0	
All care should focus on older adults' best interest/improving their quality of life	5.0		5.0		5.0		5.0		5.0		5.0	

S1 Table: Program Leader Panel Ratings of Components of a Social Inclusion Framework for an Elder Abuse Intervention

**Guiding Principle**	Expert 1	comments 1	Expert 2	comments 2	Expert 3	comments 3	Expert 4	comments 4	Expert 5	comments 5	Expert 6	comments 6
All older adults can make decisions that do not conform to social norms if no harm is done to others	4.0		5.0		5.0		5.0		5.0		5.0	
Many types of elder abuse are criminal offences	3.0		3.0		5.0		2.0		5.0		4.0	Not all types of elder abuse are criminal, but many are
There is an ethical responsibility to identify elder abuse and address it whether deliberate or inadvertent	4.0		5.0		5.0		5.0		5.0		5.0	
All care providers to older adults must be respectful of existing relationships	4.0		5.0		5.0		5.0		5.0		4.0	
Elder abuse is a complex issue	5.0		4.0		5.0		5.0		5.0		5.0	
Any intervention should empower the older adult	5.0		5.0		5.0	Create a supportive environ-ment	5.0		5.0		5.0	Create an empowering environment
All older adults have the right to clear education/ information on elder abuse	5.0		5.0		5.0		5.0		5.0		5.0	
The rights of older adults supersede the organization's/provider's personal interests	5.0		4.0		1.0		5.0		5.0		4.0	
Do no harm	5.0		5.0		5.0		5.0		5.0		4.0	

S1 Table: Program Leader Panel Ratings of Components of a Social Inclusion Framework for an Elder Abuse Intervention

**Guiding Principle**	Expert 1	comments 1	Expert 2	comments 2	Expert 3	comments 3	Expert 4	comments 4	Expert 5	comments 5	Expert 6	comments 6
Older adults are never to be held responsible for the abuse that they have experienced	5.0		5.0		4.0		5.0		5.0		4.0	
**Determinant of Health**												
Health status	5.0		5.0		5.0		4.0		5.0		5.0	
Mental capacity	5.0		5.0		5.0		4.0		5.0		5.0	
Social support	5.0		4.0		5.0		5.0		5.0		5.0	
Culture	5.0		4.0		5.0		5.0		5.0		5.0	
Socioeconomic status	5.0		4.0		1.0		5.0		5.0		3.0	
Gender	5.0		4.0		5.0	Self identified	4.0		5.0		3.0	
Religion	5.0		4.0		5.0		5.0		5.0		3.0	
Language	5.0		4.0		5.0		5.0		5.0		5.0	
Disability	5.0		4.0		5.0		5.0		5.0		5.0	
Sexual orientation	5.0		4.0		5.0		5.0		5.0		5.0	A right to sexuality, sexual beings, determinant or guiding principle?
Communication needs	5.0		5.0		5.0		5.0		5.0		5.0	
Written-in	5.0	Revictimi-zation/ trauma										

S1 Table: Program Leader Panel Ratings of Components of a Social Inclusion Framework for an Elder Abuse Intervention

**Guiding Principle**	Expert 7	comments 7	Expert 8	comments 8	Expert 9	comments 9	Expert 10	comments 10	Expert 11	comments 11	Expert 12	comments 12
All older adults have the right to self-determination (e.g., accept/refuse services, make their own decisions)	5.0		5.0		5.0		5.0		5.0		5.0	
All older adults have the right to be safe	5.0		5.0		5.0		5.0		5.0		5.0	
All older adults have the right to privacy and confidentiality	5.0		4.0		5.0		5.0		4.0		5.0	
All older adults are assumed competent unless determined otherwise	5.0		5.0		5.0		5.0		5.0		5.0	
All older adults have the right to appropriate protection (when older adult is not competent)	5.0		5.0		5.0		5.0		5.0		4.0	
All older adults have the right to dignity	5.0		5.0		5.0		5.0		4.0		5.0	
All older adults have the right to retain civil and constitutional rights (unless restricted by courts)	5.0		5.0		5.0		5.0		4.0		5.0	
All older adults have the right to informal and formal support	5.0		5.0		5.0		5.0		4.0		5.0	
All care should focus on older adults' best interest/improving their quality of life	5.0		5.0		1.0	Must be self determined	5.0		4.0		5.0	

S1 Table: Program Leader Panel Ratings of Components of a Social Inclusion Framework for an Elder Abuse Intervention

**Guiding Principle**	Expert 7	comments 7	Expert 8	comments 8	Expert 9	comments 9	Expert 10	comments 10	Expert 11	comments 11	Expert 12	comments 12
All older adults can make decisions that do not conform to social norms if no harm is done to others	5.0		5.0		5.0		4.0		5.0		4.5	
Many types of elder abuse are criminal offences	1.0		4.0		1.0	Not a principle	3.0		3.0	Some are criminal, it’s a fact, not a guiding principle	3.0	Should not be a guiding principle
There is an ethical responsibility to identify elder abuse and address it whether deliberate or inadvertent	5.0		5.0		5.0		5.0		4.0		4.0	
All care providers to older adults must be respectful of existing relationships	5.0		5.0		5.0		5.0		3.0		4.0	
Elder abuse is a complex issue	5.0		5.0		5.0		5.0		5.0		5.0	
Any intervention should empower the older adult	5.0		5.0		5.0		5.0		4.0		5.0	
All older adults have the right to clear education/ information on elder abuse	5.0		5.0		5.0		4.0		5.0		5.0	
The rights of older adults supersede the organization's/provider's personal interests	5.0		5.0		5.0		4.0		3.0		4.0	
Do no harm	5.0		5.0		1.0		5.0		5.0		4.0	

S1 Table: Program Leader Panel Ratings of Components of a Social Inclusion Framework for an Elder Abuse Intervention

**Guiding Principle**	Expert 7	comments 7	Expert 8	comments 8	Expert 9	comments 9	Expert 10	comments 10	Expert 11	comments 11	Expert 12	comments 12
Older adults are never to be held responsible for the abuse that they have experienced	5.0		5.0		5.0		4.0		4.0		5.0	
**Determinant of Health**												
Health status	5.0		5.0		5.0		5.0		5.0		5.0	
Mental capacity	5.0		5.0		5.0		5.0		5.0		5.0	
Social support	5.0		5.0		5.0		5.0		5.0		5.0	
Culture	5.0		5.0		5.0		5.0		4.0		5.0	
Socioeconomic status	5.0		5.0		5.0		5.0		5.0		5.0	
Gender	5.0		5.0	Gender identity	5.0		4.0		4.0	Gender identity	5.0	
Religion	5.0		5.0		5.0		5.0		4.0		4.0	
Language	5.0		5.0		5.0		4.0		5.0		4.0	
Disability	5.0		5.0		5.0		5.0		5.0		5.0	
Sexual orientation	5.0		5.0		5.0		3.0		4.0		5.0	
Communication needs	5.0		5.0		5.0		5.0		5.0		4.0	
Written-in			5.0	Trauma history					5.0	Revictimiza-tion		
